# Novel feature selection methods for construction of accurate epigenetic clocks

**DOI:** 10.1371/journal.pcbi.1009938

**Published:** 2022-08-19

**Authors:** Adam Li, Amber Mueller, Brad English, Anthony Arena, Daniel Vera, Alice E. Kane, David A. Sinclair

**Affiliations:** Blavatnik Institute, Dept. of Genetics, Paul F. Glenn Center for Biology of Aging Research at Harvard Medical School, Boston, Massachusetts, United States of America; University of Virginia, UNITED STATES

## Abstract

Epigenetic clocks allow us to accurately predict the age and future health of individuals based on the methylation status of specific CpG sites in the genome and are a powerful tool to measure the effectiveness of longevity interventions. There is a growing need for methods to efficiently construct epigenetic clocks. The most common approach is to create clocks using elastic net regression modelling of all measured CpG sites, without first identifying specific features or CpGs of interest. The addition of feature selection approaches provides the opportunity to optimise the identification of predictive CpG sites. Here, we apply novel feature selection methods and combinatorial approaches including newly adapted neural networks, genetic algorithms, and ‘chained’ combinations. Human whole blood methylation data of ~470,000 CpGs was used to develop clocks that predict age with R2 correlation scores of greater than 0.73, the most predictive of which uses 35 CpG sites for a R2 correlation score of 0.87. The five most frequent sites across all clocks were modelled to build a clock with a R2 correlation score of 0.83. These two clocks are validated on two external datasets where they maintain excellent predictive accuracy. When compared with three published epigenetic clocks (Hannum, Horvath, Weidner) also applied to these validation datasets, our clocks outperformed all three models. We identified gene regulatory regions associated with selected CpGs as possible targets for future aging studies. Thus, our feature selection algorithms build accurate, generalizable clocks with a low number of CpG sites, providing important tools for the field.

## Author summary

Epigenetic clocks accurately predict a person’s age by measuring the levels of a chemical mark (methylation) at specific sites of the DNA. More of these clocks are being built all the time, and there is a need for tools to best construct these clocks, and particularly to pick the specific DNA sites to include. We propose several novel machine-learning tools for the optimised selection of these DNA sites, known as feature selection approaches. We applied our approaches to a large human blood dataset to develop several clocks that accurately predict age using 35 or less DNA sites with more accuracy than previously published clocks when applied to other datasets for validation. Some of the DNA sites identified may be associated with interesting genes to explore further for their role in aging. These approaches should enable the building of more accurate, generalizable age prediction clocks from a low number of DNA sites.

## Introduction

Epigenetic clocks allow for the prediction and observation of biological aging [1]. By profiling the methylation levels at specific sites in DNA, it is possible to accurately predict the age of organisms and tissues [2]. This is often referred to as epigenetic or DNA methylation (DNAm) age. CpG sites are areas of repetitive DNA bases where a guanine follows a cytosine, which can be modified via DNA methylation and demethylation to alter the structure of chromatin and gene expression in a cell [3]. Epigenetic clocks can now predict age across multiple species and tissue types [4], and even predict mortality [5]. With the increased use of DNA methylation clocks to determine biological age and screen for interventions that slow or reverse aging, the demand for more robust, accurate clocks is growing.

The first epigenetic clocks were created by Bocklandt and colleagues [1] quickly followed by the Hannum and Horvath labs in 2013 [2,6]. The Hannum clock, based on methylation analysis of DNA from human peripheral blood mononuclear cells, was developed using elastic net regression modelling. Seventy-one markers were selected from over 470,000 CpG sites to derive an age prediction accuracy of four years [6]. Horvath’s clock encompasses multiple tissue types and includes 353 CpG sites that strongly predict age [2]. Recently, the field has focused on creating clocks with fewer CpG sites to enable epigenetic age profiling without the use of costly microarrays or expensive reduced-representation bisulfite sequencing [7,8,9,10]. Alghanim et al.’s clock, built on blood methylation data, only uses CpG sites from three gene regions to explain 84–85% of age variance [11], and Weidner’s clock based on only 3 CpG sites, is able to predict age with an error of less than five years [12].

Few epigenetic clock studies employ a discrete step to find optimal features for building clocks. In machine learning, feature selection is commonly used in situations where the number of features far outnumber the number of samples [13]. Given the vast number of CpG sites in the genome and the relatively low number of samples in most studies, feature selection methods will improve the efficiency of clock building. Currently, the most common approach for clock building is to use a ‘correlation-with-age’ method, where CpGs that have a non-zero coefficient in ElasticNet Regression analyses are given more predictive power in the model [2, 6]. The GrimAge clock selects GpGs using a Pearson correlation coefficient higher than 0.35 for further model building [5]. Some clocks utilise more advanced feature selection methods such as Boruta [14], recursive feature selection [12,15,16,17] or neural networks [9] to accurately predict age, often with few CpG sites.

There are several advantages to using feature-selection methods to build accurate clocks with fewer CpG sites. These approaches allow for the optimised identification of sites that are the most predictive of age, health and mortality from the many CpG sites that can be measured with modern technologies. As platforms become more sophisticated and the number of CpGs that are measured increases into the millions, it will become increasingly harder to select CpG sites and genomic regions of importance without sophisticated machine learning and feature selection methods. The benefits of reducing the number of features down to single digit or low double digit sites also improves accuracy. Macdonald-Dunlop and colleagues showed that for -omics based aging clocks, those with lower model complexity (built from fewer principal components) had greater accuracy [18]. While fewer CpG sites may leave the lone few features more vulnerable to confounding effects, the clocks of several hundred CpGs suffer from too much noise rather than signal for age predictions. This can be seen in the single cell context, where it was shown that including more CpGs (beyond the best top 0.5%) reduced the correlation between CpG methylation states and single cell ages [19]. Additionally, the identification of a small targeted set of CpG sites and their associated genes, allows for the focused study of these sites as biologically relevant in the study of mechanisms of aging, or possible therapeutic targets. Finally, using feature selection approaches to build low CpG clocks, will also reduce the cost of measuring epigenetic age with these clocks. The methylation status at a small number of CpG sites can be measured with less expensive targeted sequencing technologies such as TIME-seq [20] and bisulfite pyrosequencing [12], instead of relatively more costly Illumina microarrays (eg. Illumina MethylationEPIC and 450k) [21] and reduced representation bisulfite sequencing (RRBS).

Although the number of epigenetic clocks being built using feature selection is increasing, there is room to optimise feature selection methods. Here, we develop novel feature selection approaches to construct accurate epigenetic clocks with low numbers of CpG sites on the publicly available Hannum dataset (GSE40279) and evaluate their accuracy and generalizability on other datasets: GSE52588 [22], GSE137688 [23], GSE85311 [24]. We use newly adapted neural network and genetic algorithm approaches that have not previously been applied to feature selection for clocks, novel ‘chained’ combinations of standard methods, and we develop a novel upgraded selection method to optimise the construction of epigenetic clocks to predict age.

## Results

The feature selection methods selected for testing in our study include an upgraded recursive feature selection (RFE) approach, genetic algorithm, neural network feature selection via benchmark comparison, Boruta, KBest and SFM methods that are chained together for maximal performance. These present a comprehensive overview of both cutting-edge and commonly used feature selection methods. The new form of RFE, %-RFE, was created to reduce the computational power, and increase the accuracy of basic RFE on large featurespaces common in biological datasets. The advantages and disadvantages of these methods are outlined in the Methods and S1 and S2 Tables, including the details of each method and specific parameters used.

**Fig 1:**
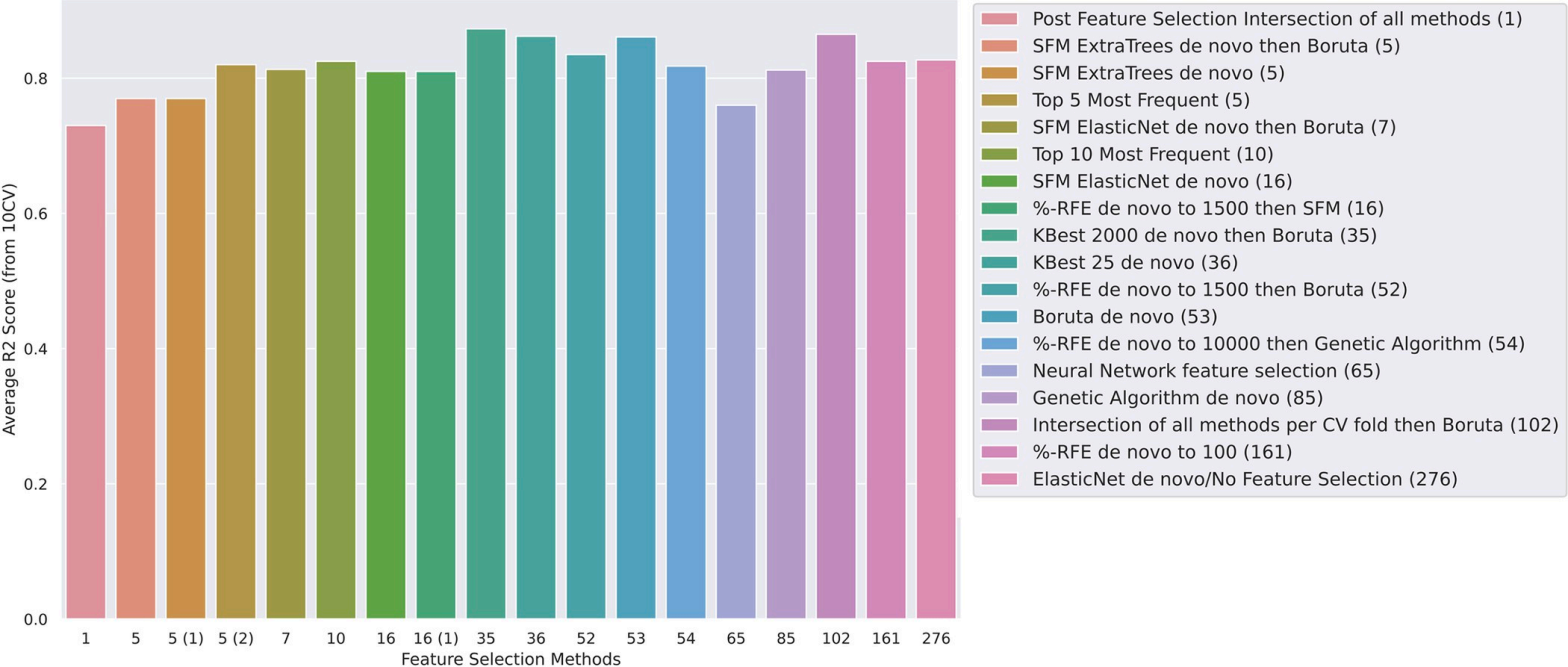
Comparative methods and the number of features used in each model on the x-axis and their average R2 scores on the y-axis. R2 scores are relatively similar across the board despite the number of features needed for prediction varying widely.

To test if accurate low CpG clocks could be built using these methods, we applied each of our feature selection approaches to the Hannum methylation dataset (GSE40279). Table 1 and Fig 1 summarise the results of the feature selection approaches, including the number of CpG sites identified with each approach, and the correlation (R2) with chronological age on a test set. The best model for age prediction for this dataset is *SelectKBest for 2000 features followed by Boruta*. This approach selects 35 CpG sites, with an R2 of 0.873 and a median absolute error of 3.08 years (Table 1).

**Table 1:** Feature selection methodology (in descending order of correlation scores). Number of features selected by each method parenthesized in the first column.

	Average R2 Score (from 10CV)	STD (Years)	Mean Absolute Error (Years)	Median Absolute Error (Years)
KBest 2000 de novo then Boruta (35)	0.873	0.05	3.82	3.08
Intersection of all methods per CV fold then Boruta (102)	0.865	0.06	3.9	3
KBest 25 de novo (36)	0.862	0.06	3.96	3.14
Boruta de novo (53)	0.861	0.06	3.95	3.08
%-RFE de novo to 1500 then Boruta (52)	0.835	0.07	4.35	3.57
ElasticNet de novo/No Feature Selection (276)	0.827	0.06	4.64	3.91
%-RFE de novo to 100 (161)	0.825	0.07	4.69	3.83
Top 10 Most Frequent (10)	0.825	0.08	4.59	3.7
Top 5 Most Frequent (5)	0.82	0.08	4.6	3.79
%-RFE de novo to 10000 then Genetic Algorithm (54)	0.818	0.08	4.61	3.76
SFM ElasticNet de novo then Boruta (7)	0.813	0.07	4.7	3.71
Genetic Algorithm de novo (85)	0.812	0.08	4.72	3.68
SFM ElasticNet de novo (16)	0.81	0.07	4.74	3.84
%-RFE de novo to 1500 then SFM (16)	0.81	0.07	4.74	3.84
SFM ExtraTrees de novo (5)	0.77	0.08	5.36	4.27
SFM ExtraTrees de novo then Boruta (5)	0.77	0.08	5.36	4.271
Neural Network feature selection (65)	0.76	0.08	5.65	4.79
Post Feature Selection Intersection of all methods (1)	0.73	0.09	5.75	4.38
Variance Threshold de novo (2)	0.02	0.02	11.9	10.61

*ElasticNet de novo* (Table 1 and Fig 1) represents a model without any feature selection methods for comparison to the other models. This model uses all ~450,000 features to train a model without any pre-selection or iterative algorithms. The resulting clock from this approach is based on 276 CpGs, which is an order of magnitude more CpGs than clocks developed with the feature selection methods (Table 1), and with a lower R2 score than five of the feature selection models (Table 1).

All of our novel updated %-RFE methods worked well with scores of 0.81 or higher (Table 1). Several of our combinatorial ‘chained’ approaches also performed well, in particular *KBest 2000 de novo then Boruta* which was the highest scoring clock (R2 = 0.87) and *%-RFE de novo to 1500 then Boruta*, which had an R2 of 0.835. *Boruta de novo* and *KBest 25 de novo* score remarkably well with no prior method being applied (0.861 and 0.862 respectively). These are the best performing solo feature selection methods.

Our other feature selection methods, including most of the SelectFromModel (SFM) methods and the genetic algorithms, achieve an accuracy of between 0.77 to 0.81 (Table 1). Despite being fundamentally different in their approach, these methods accomplish similar results and plateau in the same range of scores (Fig 1). Further optimization of each of these methods is needed to warrant their usage over other more successful methods.

Using the five most frequently selected CpGs among all the methods to build a clock resulted in a correlation score of 0.83 and median absolute error of 3.79 years (Table 1). Table 2 shows the corresponding GeneIDs for these CpGs. The most frequent CpG site is cg16867657 (ELOVL2) and training a clock on this single feature results in a correlation score of 0.73 (Table 1). Overall, these results demonstrate that using feature selection methods accurate epigenetic clocks can be constructed with only a few CpGs. We also provide a table of all CpGs included in all of our clock models (S3 Table). These sites, and their associated genes may be novel markers or targets for future age-related research.

**Table 2:** The five CpG sites that are chosen as most frequent predictors for aging and their associated gene symbols.

Most Frequent 5 CpG Sites	Associated GeneID
cg16867657	ELOVL2
cg10501210	C1orf132
cg22454769	FHL2
cg04875128	OTUD7A
cg19283806	CCDC102B

We also tested a neural network approach for feature selection. An ElasticNet Regression model trained on the top 65 features selected by the neural network, has a moderate R2 value of 0.76. Interestingly, only four of the 65 identified neural network CpGs overlap with the CpGs selected by other methods described here.

**Fig 2:**
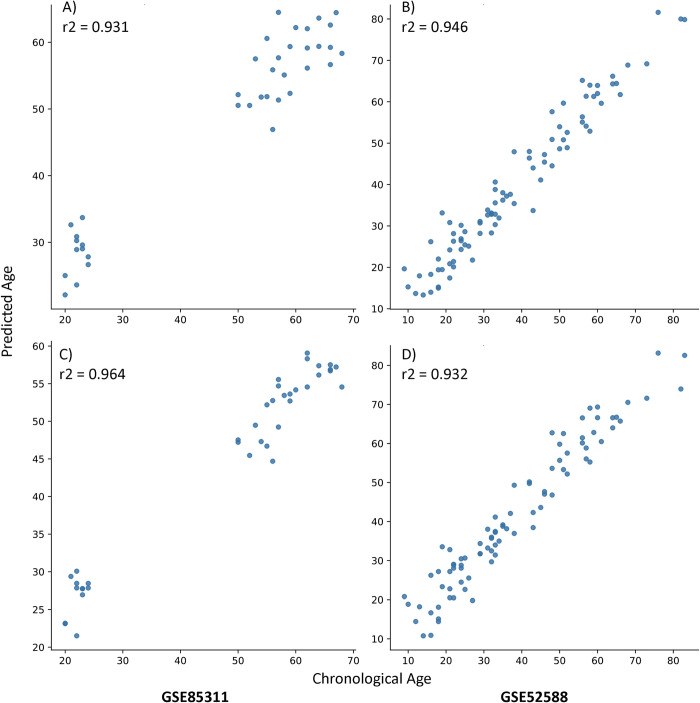
Figure showing the Predicted Ages vs Chronological Ages from our two final models on the two external validation datasets GSE85311 and GSE52588. (A-B) KBest 2000 de novo then Boruta (C-D) Top 5 Most Frequent.

We selected two models developed above for further validation of their accuracy in independent datasets. *SelectKBest for 2000 features followed by Boruta* & the *top 5 most frequent features* are the best performing feature selection method and the clock with the lowest number of CpGs sites, respectively. We applied these two clock models to two published blood methylation datasets. GSE85311 contains methylation profiling of blood taken from young and old human subjects of varying exercise level [24]. GSE52588 contains methylation profiling of blood taken from people with and without down syndrome [22]. Each of the clocks predicted age very well in these external data sets with R2 values greater than 0.93 (Table 3 and Fig 2).

**Table 3:** Table showing the results of the two final models trained on the Hannum dataset (GSE40279) [6]) validated on external datasets: Horvath down syndrome blood dataset (GSE52588)[22], Martens exercise blood dataset (GSE85311)[24], and buccal dataset (GSE137688)[23]. Number of CpG sites/features in parentheses.

Feature Selection Methods	Data set	R2 Score	Mean Absolute Error (Years)	Median Absolute Error (Years)
KBest 2000 de novo then Boruta (35)	GSE85311	0.931	4.66	4.18
	GSE52588	0.946	3.35	2.68
	GSE137688	0.710	2.0	1.6
Top 5 Most Frequent (5)	GSE85311	0.964	5.71	5.60
	GSE52588	0.932	4.56	3.98
	GSE137688	0.470	2.72	2.29

**Fig 3:**
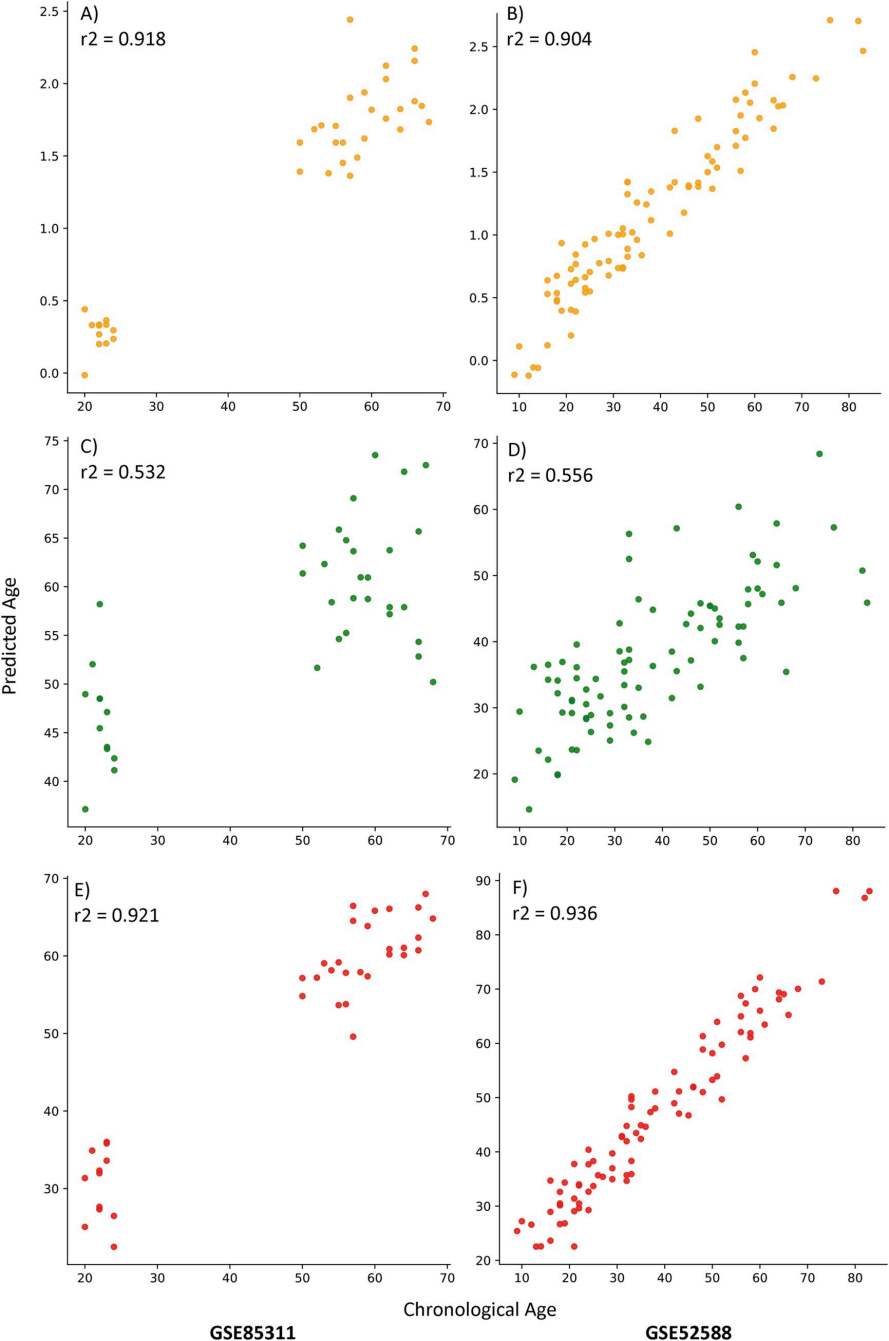
Figure showing the Predicted Ages vs Chronological Ages from Horvath’s, Weidner’s and Hannum’s publicly available models/equations on the two external validation datasets GSE85311 and GSE52588. (A-B) Horvath’s model (C-D) Weidner’s model (E-F) Hannum’s model.

We also compared the performance of our top two clocks against previously published clocks by Horvath (one of the gold standard benchmark models in the epigenetic clock field, 353 CpGs) [2], Weidner (one of the lowest published CpG clocks, 3 CpGs) [12] and Hannum (created from the same dataset as our clocks, 71 CpGs) [6]. These 3 clocks were applied to the same datasets as above (GSE85311 andGSE52588) to predict age. As is shown in Fig 3, our models had higher age correlation coefficients than all three of these previously published clocks in the prediction of age in both of these datasets.

To test whether clocks developed with our feature selection approaches can be applied to datasets taken from other tissue types, we applied our two selected best models to a human buccal cell dataset (GSE137688) [23]. Using the methods on this dataset, we achieved a top R2 score of 0.71 with the *SelectKBest for 2000 features followed by Boruta* method and R2 of 0.47 with the *Top 5 Most Frequent* method (Table 3). The scores were expectedly lower than the results of the previous two validation sets because the clocks were trained on blood data, and applied to buccal swab data, which have inherent sampling and variance differences. While the R2 scores were not as high, the models did have very low mean and median absolute errors; the lowest of all results in this paper. Given the abundance and ease of accessing buccal samples, this represents promising rudimentary groundwork for the application of feature selection methods on sample types beyond blood.

We next wanted to test whether the features selected with our methods could be used to make accurate clocks in other datasets. We took the CpGs selected from the Hannum dataset using our top two models (*SelectKBest for 2000 followed by Boruta* & the *Top 5 Most Frequent* CpG features), and selected those same CpGs in the Horvath down syndrome dataset (GSE52588) [22]. Using only those CpGs, we created a clock from that remaining dataset using the same cross-validation scheme (see Methods) used for our original Hannum experiment above. Remarkably, the clocks developed in this dataset based on 35 features (*SelectKBest for 2000 features followed by Boruta*) and 5 features (*top 5 most frequent*) achieved R2 scores of 0.928 and 0.911 respectively (Table 4), indicating that these CpGs can be selected across datasets to create accurate clocks and are possibly universal CpGs for predicting age.

**Table 4:** Results of the two models created from the Horvath down syndrome blood dataset (GSE52588) [22] using the same CpGs selected from the two feature selection methods from the initial Hannum experiment. These models were validated using the same 10CV scheme from the initial Hannum experiment. Number of CpG sites/features in parentheses.

Feature Selection Method CpGs used	Average R2 Score (from 10CV)	Mean Absolute Error (Years)	Median Absolute Error (Years)
KBest 2000 de novo then Boruta (35)	0.928	3.39	2.92
Top 5 Most Frequent (5)	0.911	4.02	3.72

## Discussion

Overall, we demonstrate that feature selection methods can select CpG sites that are highly predictive of age, allowing for less features needed to build highly accurate epigenetic clocks. Our two best clocks were validated on external datasets, and in fact out-performed previously published epigenetic clocks. However, many different types of feature selection methods, including novel optimised and combinatorial methods, are able to attain a reasonably high correlation score of around 0.75–0.85 whilst using a low number of CpG features. Developing these accurate low CpG clocks allows for the focused investigation of these sites and cheaper measuring of epigenetic age. Our rudimentary base code that outlines most of the feature selection ideas in this paper is publicly available and we hope that feature selection becomes a standard discrete step in future epigenetic clock studies.

When our two best clocks were applied to validation datasets both performed well, especially in comparison to clocks by Horvath, Hannum and Weidner [2,6,12]. The benefit of our methods over both Hannum and Horvath’s clocks are higher predictive accuracy and fewer CpGs meaning less costly future epigenetic age profiling. Weidner’s clock has two fewer CpGs than our smallest clock, but it does not predict age as accurately on the two validation sets. Interestingly, Weidner and colleagues used a standard RFE feature selection approach in the development of their clock.

For further validation of our approaches, we also applied the two best models to a dataset of a different sample type; buccal epithelial cells. Although the R2 scores were only moderate for this dataset, the mean and median absolute errors were the lowest we observed. This suggests an interesting future potential for buccal/saliva methylation samples, as they are much more accessible and less expensive to obtain. The moderate performance of our blood-based clock on buccal cell samples, also highlights the importance of having appropriately trained clocks for the outcomes and samples you wish to measure. As shown by Liu and colleagues, DNA methylation is global, but it is not uniform [25]. They compared the CpGs of 11 well known epigenetic clocks and found little overlap in their genomic locations [25]. As DNA methylation is heterogeneous across sources and contexts, it is important to have readily available tools, such as those presented here, that can select for the optimal sets which predict best the target variable.

The feature selection methods presented here represent novel steps forward in the field. Firstly, we present two algorithms (the neural network and genetic algorithm) that were designed and written without modification of existing packages. To the best of our knowledge, this is the first application of these methods to feature selection for aging biomarkers, and their application in general biology is nascent at best. Although the neural network was not as accurate as the other feature selection methods, it did select many CpG features that were missed by our other approaches. We conclude that this is a promising predictive tool to uncover more obscure CpGs that most conventional methods miss. Furthermore, unlike the other methods such as RFE and Boruta, the neural network selection method has substantially more parameters one can optimise for in different contexts, including number of nodes, cost function, activation function and number of hidden layers, but also setting the target CpG site’s methylation (see Methods) to a range of values, instead of just 0 and 1, and ranking the importance of features based on the fluctuation of the score given a threshold of methylation. As methylation datasets become larger in sample size and more sophisticated neural network models are developed, their performance can only improve. Genetic algorithms were initially created for model or parameter selection but in recent years have become one of the most advanced feature selection methods in computer science [26]. Our genetic algorithm performed moderately well in the selection of features to predict age, and is designed to offer the user several options for parameter tuning at different stages such as choosing how many models or ‘creatures’ to populate with, mutation rates, mating habits etc. (see Methods for more details). The avenues for parameter optimization in genetic algorithms is truly vast and we have only showcased genetic algorithms ability to keep up with older established feature selection methods in predicting age. However, combined with its heavy potential for hyper-specific parameter tuning, it gives promise to possibly surpass them in the future.

In addition to the neural network and genetic algorithm, we also present novel adaptations of standard feature selection libraries. These include an upgrade to the conventional RFE that enables the sklearn libraries to both run more efficiently and produce better results, as well as ‘chaining’ different feature selection methods together, in order to cover each other’s weaknesses to produce a more well-rounded feature selection pipeline. The latter produced our best performing model (*SelectKBest method down to 2000 features followed by Boruta*), demonstrating that chaining these methods can result in novel and superior feature selection approaches. Our modification to the RFE approach, although small, is significant and perhaps necessary when it comes to feature selection in large datasets. Whilst the stock RFE package is only capable of removing a user set number of features regardless of its relation to the current feature space size, our novel %-RFE removes a target number of features that scales dynamically with every iteration of the algorithm. This enables the algorithm to be computationally less intensive, but also possibly produce better results (Table 1). Boruta also suffers from a similar computational issue as it essentially doubles the dataset (see Methods) to create its shadow features for feature selection. Decision trees and random forests are also its main model architectures, models which take much longer to train compared to regression models. In order to fully complete a stock Boruta feature selection run, a reduced number of trees must be used to reduce runtime (S1 Table), but this low number of trees can affect the training capacity. Here, we use %-RFE or other feature selection methods in tandem with Boruta to first reduce the dimensionality and drastically improve its performance. RFE and Boruta has been used prior to this study in the creation of epigenetic clocks [12, 14, 15, 16, 17], however the novel combination of these methods together allow users to use these models to their full potential in the context of methylation data where the number of features vastly outnumber samples. This study validates the concept of using several feature selection methods in tandem with each other to overcome computational issues and still achieve competitive results.

In this study, we identified five CpGs and their corresponding genes that were of particular interest, as they were most commonly identified across all feature selection methods in our study (Table 3). Four of these CpG sites, and particularly ELOVL2, have been previously identified as strong predictors of age. ELOVL2, C1orf132, FHL2 and CCDC102B are included in an online seven CpG site epigenetic clock from the University of Santiago de Compostela [27]. Zbieć-Piekarska et al constructed a linear regression model using only ELOVL2’s CpG site (cg16867657) to predict age [28] and obtained a high degree of accuracy in blood samples from humans. By manipulating the expression of ELOVL2 and observing age-related changes in the eyes of mice, [29] suggest that the gene is a molecular regulator of aging in the retina. Spólnicka and colleagues used ELOVL2 to accurately detect age differences from 3 disease groups [9], and also highlight C1orf132 and FHL2 as key genes from which CpG sites are used for their epigenetic clock. CCDC102B is also linked to aging and age-related degenerative diseases [30,31]. Ito and colleagues developed a clock using only the CpG sites associated with CCDC102B and ELOVL2 [7] and can predict age with an R2 of 0.75. Additionally, Fleckhaus et al.’s study develops a clock using 8 target regions, four of which are ELOVL2, FHL2, CCDC102B and C1orf132 [32]. These papers show that our feature selection methods are able to select the most age predictive CpG sites, consistently with other studies. OTUD7A is the fifth gene of interest that we identified with our methods and the least documented. One study identified highly methylation CpG sites associated with OTUD7A correlating with age [33], and Yin et al. identified it as a potential regulator for neurodevelopmental disorders [34]. The role of OTUD7A in aging, if any, is not well-known and should be explored further. In addition, 61 of the CpG sites identified by our neural network analysis had no overlap with other clock sites selected by us or Hannum’s model in the original Hannum dataset and may provide novel biologically important targets. We hope that the application of these feature selection methods in other studies and across more datasets by our group and others, will allow the future identification of more novel age-related genes.

In the future, these methods can be applied to a range of studies developing epigenetic clocks including across new tissue types (such as buccal/saliva samples), or by examining a limited subset of CpGs in mutual overlap between bulk methylation and single cell datasets [19]. Parallelized, highly cost-reduced methods targeting specific CpG regions, such as TIME-seq [20] will dramatically lower the cost barrier of entry into epigenetic clock analysis. As Illumina arrays cost several hundred dollars a sample, the combination of our feature selection methods that find low amounts of important CpGs and cheaper sequencing approaches will prove to be a powerful combination. Lastly, these methods are not limited to the identification of CpG sites as features, and this pipeline could be used to identify features for biomarkers or clocks developed from a range of datasets (eg. metabolomics, microbiome, transcriptomics, proteomics, clinical data), and to predict a variety of age and health outcomes. Given the vast feature space of -omics datasets, creating an accurate model through penalised regression is often not difficult, however finding the right features for further study to infer biological understanding is harder. In recent years, feature selection has become a popular method for novel biomarker discovery [35, 36, 37, 38, 39, 40] and the application of the novel feature selection methods in this paper could accelerate the discovery of biomarkers in many fields.

## Methods

### Data

The datasets for this study are from the Gene Expression Omnibus database under the accession codes GSE40279, GSE85311, GSE52588 and GSE137688 [6,22,23,24]. These datasets were pre-processed from raw methylation data and provided by their respective authors and studies. To ensure our models and publicly available models were compared fairly during validation we removed the CpG sites with missing methylation data across all clocks and datasets. The main dataset GSE40279 we test the feature selection methods on contains 656 samples (instances) of whole blood human methylation levels at 473,035 CpG Sites (features), matched to chronological ages. All analysis was done in Python 3. All related code outlining our methods is available on github (https://github.com/adamyli/CLK-MKR).

### Cross-validation and overall approach

**Fig 4:**
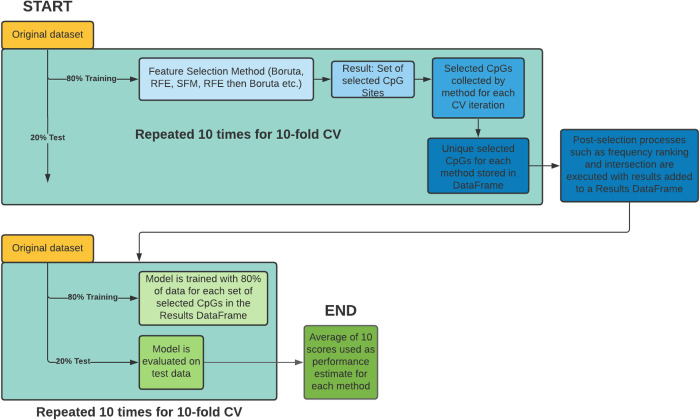
The workflow for feature selection and model evaluation. Feature selection was performed on training data for each iteration of 10-fold cross validation. The selected features of each iteration are aggregated into a list for each feature selection method type. The unique selected features for each method are collected into a dataframe where post-selection processes such as intersections are performed. We add the results to a dataframe. Each column of selected features in the results dataframe (each representing a different feature selection method) is tested using another training-testing split on the original data. This is done 10 times for 10-CV with the average of all scores being the performance estimate for that feature selection method.

The main workflow methodology is outlined in Fig 4. The original dataset was split into 10 folds for cross-validation (CV). For each set of training folds, every different feature selection method was performed to select the optimal features within that training data. For every CV iteration, the intersection of each feature selection method was also recorded and we performed Boruta on the intersected features. For each of the feature selection methods, unique features from each of the 10 iterations were collected into an aggregated list and entered into a final results dataframe. This dataframe contains every unique feature selected by each selection method at each of the 10 iterations.

Post-feature selection processes were then performed. These include the intersection between the results of all selection methods and ranking the top 5 and 10 most common features out of all the results. The results from these two post-feature selection processes were also added to the Results Dataframe. The original dataset was split into 10 folds again and for each column of the Results DataFrame, which represents the unique selected features for every method, we reduced the dataset down to the selected features. We trained the ElasticNet regression model for chronological age using training data (80%) and evaluated the model on the test data (20%) using the r2 scoring metric. For each column the mean of the 10 r2 scores was the performance estimate of that feature selection method.

The best performing model was the clock from the *SelectKBest method down to 2000 features followed by Boruta* resulting in 35 selected features. The second model of interest uses the top 5 most frequently selected CpGs. These 2 models were validated using two external blood methylation datasets; (GSE52588) and (GSE85311) and their performance was compared to Hannum’s model’s predictions on these two datasets. The features from these two models were also used to build models from the GSE52588 dataset and predict age using the same 10-fold CV as the Hannum dataset to investigate if these selected features are effective across datasets.

These two models are also applied to a methylation dataset taken from buccal cells (GSE137688) to see if performance could be replicated in conventionally cheaper samples [23].

### Feature selection methods

#### SelectFromModel (SFM)

SFM is a function within skLearn [41] that wraps around and trains a model on a dataset and allows the user to specify a threshold of feature importance. Depending on whether the model is a standard regression or random forest model, the feature importance is calculated from the coefficients or mean importance respectively. Features (CpG sites) with less feature importance than this threshold are discarded, leaving only the features with the highest coefficient or importance. This method is fast but simple. Thresholds of 0.01, 0.05, 0.1, 0.5 are tested as thresholds beyond this point yielded 0 features for every input dataset and finer intervals between each threshold had minimal changes in number of features selected. For this study, the models that the SFM wraps around are ElasticNet Regression and ExtraTrees forest.

The ExtraTrees Regression estimator is composed of a number of decision trees. A decision tree can be thought of as an intuitive flowchart where an answer to one decision between 2 or more choices leads to another. Decision trees decide how to split by prioritizing the split that creates the least uniform distribution of labels or values. This branching of nodes continues until it reaches a node that cannot decide which split to use because they result in equally uniform distribu-tions—meaning any more branches will not help the tree make any better decisions. In this sense ExtraTrees is similar to the more popular random forest with a few distinct differences. Random Forest samples the training data with replacement to train their decision trees whileExtraTrees uses the entire original dataset. However ExtraTrees randomly chooses the split instead of optimally finding a locally one which is what Random Forest does. ExtraTrees are therefore less exhaustive in their optimization and are faster than Random Forests. This is ideal for us as a Random Forest with 5–8 trees in it can take several hours to train on a dataset as large as ours. A random forest takes an advantage known as bagging by taking random instances of the dataset and training its model from solely those samples. For a regression problem like ours the average value of all trees are taken as the final prediction.

#### Recursive Feature Elimination (RFE) and the introduction of %-RFE

RFE is a function that trains a model on a dataset and removes the weakest feature based on the lowest feature importance from the dataset [41]. This new dataset of N-1 features is trained again with a model and the process is repeated until only the user specified number of features is left. By removing 1 feature each time, RFE is a brute force algorithm that leaves only the best performing features at each iteration. However it does not take into account all features at the same time, and is unable to be aware of relationships between CpGs when it comes to predicting age e.g. some CpGs may become a strong predictor of ageing in the presence or absence of another.

Applying the stock RFE algorithm to our dataset of 473,035 features is computationally limiting due to the size of the dataset (S1 Table). Instead, we wrote a novel algorithm that removes a percentage-based number of features at each iteration allowing us to aggressively remove the majority of unnecessary features at the start but be more meticulous with our selection near the end. The percentage chosen is 1%, i.e. removing 4730 features at 473,035 and 1 feature at 100. Depending on our use case we used %-RFE down to 100, 1500 and 10,000 features. This variation allowed us to feed different amounts of features into more computationally intensive but higher quality feature selection methods.

#### Boruta

RFE is a ‘minimal optimal’ feature selection method, meaning it attempts to select the smallest set of features with the minimum error for an estimator and aims to optimize this ratio. Boruta differs as an ‘all-relevant’ feature selection method compatible with only tree-based regression methods, such as random forests[42]. Instead of trying to find the most compact set of features to predict with, it considers all features that could possibly contribute towards prediction overcoming the weakness of RFE’s greedy nature. Boruta creates duplicates of the existing features with randomized values called ‘shadow features’. The dataset comprising the original and the shadows, is trained on the tree estimator and the shadow features compete with their original forms. Features that consistently beat their shadow counterparts are selected as reputable predictors. In order to deal with the computational power needed to train a random forest with over 470,000 features, we used only 7–8 trees and 100 iterations when using Boruta de novo on all ~470,000 features. When using Boruta with other feature selections later on (after a faster method was applied) the default number of trees and iterations was able to be used.

#### SelectKBest

SelectKBest is a feature selection method in sklearn similar to SFM that fits a dataset and selects features based on a scoring metric [41]. For each feature it calculates the correlation value between the feature and target label and ranks them. This method is fast due to its shallow nature of only training once so is not useful when used alone. However, it is helpful to reduce the total number of features for usage of more greedy algorithms such as Boruta. In our methodology we select the top 25 features and the top 2000 features using SelectKBest. We perform Boruta on the top 2000 features.

#### Variance threshold

Variance threshold is a simple and exploratory method that removes all features whose column of values do not reach the threshold of variance [41]. Since some datasets naturally may not have a high degree of variance in their recorded data, this method is not consistent. However since its execution is the fastest out of all the methods (S1 Table) it is included as an added method.

#### Neural Network (NN) feature selection

The rudimentary neural network is built using PyTorch to feature select CpG sites, as neural networks have been known to capture nonlinear relationships between data points. We were interested in seeing what would be good predictors of aging that might have been missed by the other linear regression models and lay the groundwork for future feature selection using NNs. As a proof-of-concept we used %-RFE to reduce the number of features from 473,035 down to 100. The NN first uses all 100 original features and trains the model once, its score being recorded as a benchmark. Following this, for each of the 100 features, the NN is then trained twice; once where all methylation levels of that feature equals 1 and once where they all equal 0 to simulate the CpG being fully methylated and also absent. Both are done to account for the cases where the original methylation value is close to 0 or 1. The mean of the two resulting scores are compared to the benchmark with the difference being recorded for each CpG site. The CpG sites are ranked in difference to establish an idea of feature importance with the postulation that a larger difference between the presence and absence of the CpG will insinuate that the CpG has a greater impact on age prediction. The top 50–75 are recorded as selected features.

#### Genetic algorithm

An algorithm based on the nature of Darwinism evolution where a population of ‘creatures’ are assigned a desired amount of features from the original dataset at random. These creatures are evaluated via predicting a validation set and assigned a score or ‘fitness’. The lowest scoring creatures are culled next, simulating survival of the fittest. The remaining creatures are bred by creating a child creature that has features from their shared ‘gene pool’ and having a new number of them selected randomly. There is a chance for a certain number of these ‘genes’ to be mutated. Meaning some of the features will be randomly swapped for a different one from the original dataset. This helps introduce variation. This process is repeated for a specified number of generations or until a desired fitness is met.

The genetic algorithm is powerful as it allows the user many points of optimization, depending on the creativity of the user. For instance, the number of generations, number of features and creatures are all linked variables where a perfect balance can be found. When it comes to the breeding process it is possible to implement a ‘polygamous’ aspect where a highly successful creature is allowed to breed multiple times to ensure the most predictive features are passed on and tested further in other combinations. Mutation rate, number of genes allowed to mutate as well as number of children produced per breed (with possibility of scaling number of children produced with the fitness of the parent). It is also common for genetic algorithms to be run in parallel, predicting subsets of a label, e.g. an algorithm for young samples and one for old. For our model we used 50 features per ‘creature’ model and 3000 creatures in our population. After each epoch we culled 50% of the population and mutate 30% of their features on a random chance. These parameters were chosen to ensure a rapid ‘evolution’ of the population to speed up selection time by culling half each time. The mutation rate was kept random but when it did happen we ensured that a sizeable block of features were changed so we could keep introducing variation.

### Novel methods combining multiple feature selection methods

The introduction of %-RFE allows us to synthesize novel feature selection methods. %-RFE allows for the removal of ‘fluff’ down to a more manageable number of features (usually a few thousand) and allows for more powerful methods to be used such as Boruta, Neural Networks and RFECV. These methods require more iterations and computational power so being able to distil down to the most important thousand features to choose from is ideal. The synthesized methods consist of %-RFE first selecting features to an amount appropriate for the next method. SFM is also used as a preliminary selection method in this way. The final synthesized methods consist of modular code functions that allow us to alternate the order in which the selection methods are used as well as let us combine them together and use the output of one method as the input of another.

### Clock models

The epigenetic clocks are built using ElasticNetRegression models. ElasticNet is chosen as it is the current standard for epigenetic clocks and outperforms Random Forests and SVMs with these data and feature selection methods.

This model is a variant of classical linear regression. This aims to solve for the coefficients of a linear equation that equals the ‘best fit line’. The best fit line minimizes the sum of squares by having the least distance between the data points and the line. The equation for ordinary linear regression is as follows:

$$\mathrm{argmin}=\sum {\left(y_{a}-y_{p}\right)}^{2}$$

$$\mathrm{argmin}=\sum {\left(y_{a}-(\beta_{1}x_{1}+\ldots \beta_{n}x_{n})-b\right)}^{2}$$

Where y_a is the actual value of the target label and prediction y_p calculated by the summation of predictors ‘x’ multiplied by a vector of coefficients β_n that is found from fitting the model b.is the y-intercept. argmin signifies a cost function where we seek to minimize the answer given input arguments.

Regularization is a process in which different variants of bias and penalties are introduced to assist in finding the solution to this equation that allows for the best predictive accuracy. These penalties are controlled by a lambda value (alpha in sklearn) that controls how heavy (large) this penalty is. The L1 penalty is referred to as Lasso Regression, it adds a bias that is the absolute value of the coefficients. The L2 penalty is referred to as Ridge regression, this adds a bias that is the squared value of the coefficients. Unlike ridge regression, lasso regression can shrink the coefficients of unneeded parameters (features) to 0 (due to the penalty term not being squared), essentially eliminating them, leaving only useful features. Lasso can be quite aggressive however, taking only 1 feature out of several correlated ones or selecting too few. This is where ElasticNet comes in. The generic form of the ElasticNet equation is:

$$\mathrm{argmin}=\sum {\left(y_{a}-\beta x_{n}\right)}^{2}+\lambda_{1}\sum |\beta |+\lambda_{2}\sum \beta^{2}$$

Where L1 is the regularization penalty for the ‘Lasso’ part of the regression equation andL2 is the penalty for the ‘Ridge’ portion [43]. ElasticNet combines both Lasso and Ridge regressions, adding both terms to the equations. Each penalty gets an independent alpha / lambda that is tuned via cross-validation or other methods. This method allows the best of both worlds depending on the feature.

## Supporting information

S1 Table Results from initial testing of computational runtimes for the exhaustive feature selection methods. (DOCX)

S2 Table Overview of advantages and disadvantages between different feature selection methods. (DOCX)

S3 Table CpGs, and associated genes where available, for all clocks developed using the different feature selection methods. (CSV)
