# Supplementary material for: Novel feature selection methods for construction of accurate epigenetic clocks
Source: PLoS Comput Biol. 2022 Aug 19;18(8):e1009938. doi: 10.1371/journal.pcbi.1009938 (PMC9432708; doi:10.1371/journal.pcbi.1009938)
Supplement: S1 Table — (DOCX) [file pcbi.1009938.s001.docx]

| **Feat. Selection Method** | **Time** | **Notes** |
| --- | --- | --- |
| SFM (Iterative Scoring) | ∼10 Minutes | Very few to no features selected |
| SFM (Reduction to 7 features) | ∼4 Hours | Very few to no features selected |
| RFE ElasticNet | ∼24 Hours (No Completion) | ∼10,000 iterations before termination |
| RFE Random Forest | ∼4 Hours (No Completion) | 26 iterations before termination |
| Boruta (100 Iterations/’Auto’ Estimators) | ∼25 Hours (No Completion) | 0 iterations completed |
| Boruta (100 Iterations/100 Estimators) | ∼7 Hours (No Completion) | 1 iteration completed |
| Boruta (100 Iterations/50 Estimators) | ∼3 Hours (No Completion) | 1 iteration completed |
| Boruta (100 Iterations/25 Estimators) | ∼10 Hours (No Completion) | 1 iteration completed |
| Boruta (100 Iterations/10 Estimators) | ∼4 Hours (No Completion) | 4 iterations completed |
| Boruta (100 Iterations/7 Estimators) | ∼4 Hours | 28 Features Chosen |
| Boruta (100 Iterations/5 Estimators) | ∼3 Hours | 12 Features Chosen |
| Boruta (100 Iterations/2 Estimators) | ∼1 Hours | 5 Features Chosen |
| Boruta (2 Iterations/2 Estimators) | ∼9 Minutes | 0 Features Chosen |

**S1 Table.** Results from initial testing of computational runtimes for the exhaustive feature selection methods
