# Supplementary material for: Novel feature selection methods for construction of accurate epigenetic clocks
Source: PLoS Comput Biol. 2022 Aug 19;18(8):e1009938. doi: 10.1371/journal.pcbi.1009938 (PMC9432708; doi:10.1371/journal.pcbi.1009938)
Supplement: S2 Table — (DOCX) [file pcbi.1009938.s002.docx]

| **Name of Method** | **Advantage** | **Disadvantage** |
| --- | --- | --- |
| SFM | Very fast | Not exhaustive in selection, must be done iteratively to select |
| %-RFE | Highly exhaustive | Difficult to decide optimal stopping point |
| Boruta | Highly exhaustive | Computationally extensive |
| SelectKBest | Very fast | Not exhaustive in selection, must be done iteratively to select |
| Neural network benchmarking | Can uncover missed important features | Computationally intensive in this current study |
| Genetic Algorithm | Exhaustive, can be done iteratively ad infinitum, highly customizable | High number of parameters can take a long time to optimise |
| SelectKBestfollowed by 2nd method | Extremely quick and exhaustive and the best performing in this study | Needs iteration to optimise final KBest number of features |
| %-RFE followed by 2nd method | The most exhaustive method in this study | Computationally intensive if trying to optimise number of features |
| SFM followed by 2nd method | Extremely quick and exhaustive | SelectKBest |
| Variance Threshold | Very fast | Statistical test was not applicable to this study |

**Supplementary Table 2.** Overview of advantages and disadvantages
